# *LIM only 4 *is overexpressed in late stage pancreas cancer

**DOI:** 10.1186/1476-4598-7-93

**Published:** 2008-12-22

**Authors:** Jun Yu, Kenoki Ohuchida, Kohei Nakata, Kazuhiro Mizumoto, Lin Cui, Hayato Fujita, Hiroshi Yamaguchi, Takuya Egami, Hidehisa Kitada, Masao Tanaka

**Affiliations:** 1 Department of Surgery and Oncology, Graduate School of Medical Sciences, Kyushu University, Fukuoka, Japan; 2 Department of Integration of Advanced Medicine and Innovative Technology, Graduate School of Medical Sciences, Kyushu University, Fukuoka, Japan; 3 Department of Anatomical Pathology, Graduate School of Medical Sciences, Kyushu University, Fukuoka, Japan; 4 Department of Anatomical Pathology, Saitama Medical University International Medical Center, Saitama, Japan

## Abstract

**Background:**

LIM-only 4 (LMO4), a member of the LIM-only (LMO) subfamily of LIM domain-containing transcription factors, was initially reported to have an oncogenic role in breast cancer. We hypothesized that LMO4 may be related to pancreatic carcinogenesis as it is in breast carcinogenesis. If so, this could result in a better understanding of tumorigenesis in pancreatic cancer.

**Methods:**

We measured *LMO4 *mRNA levels in cultured cells, pancreatic bulk tissues and microdissected target cells (normal ductal cells; pancreatic intraepithelial neoplasia-1B [PanIN-1B] cells; PanIN-2 cells; invasive ductal carcinoma [IDC] cells; intraductal papillary-mucinous adenoma [IPMA] cells; IPM borderline [IPMB] cells; and invasive and non-invasive IPM carcinoma [IPMC]) by quantitative real-time reverse transcription-polymerase chain reaction (qRT-PCR).

**Results:**

9 of 14 pancreatic cancer cell lines expressed higher levels of *LMO4 *mRNA than did the human pancreatic ductal epithelial cell line (HPDE). In bulk tissue samples, expression of *LMO4 *was higher in pancreatic carcinoma than in intraductal papillary-mucinous neoplasm (IPMN) or non-neoplastic pancreas (*p *< 0.0001 for both). We carried out microdissection-based analyses. IDC cells expressed significantly higher levels of *LMO4 *than did normal ductal epithelia or PanIN-1B cells (*p *< 0.001 for both) or PanIN-2 cells (*p *= 0.014). IPMC cells expressed significantly higher levels of *LMO4 *than did normal ductal epithelia (*p *< 0.001), IPMA (*p *< 0.001) and IPMB cells (*p *= 0.003).

**Conclusion:**

Pancreatic carcinomas (both IDC and IPMC) expressed significantly higher levels of *LMO4 *mRNA than did normal ductal epithelia, PanIN-1B, PanIN-2, IPMA and IPMB. These results suggested that *LMO4 *is overexpressed at late stages in carcinogenesis of pancreatic cancer.

## Introduction

Pancreatic cancer is one of the most aggressive malignant tumors. It is the fifth leading cause of cancer death in Japan [1,2] and has the lowest survival rate of any solid cancer [3]. Because the lack of specific symptoms in patients with pancreatic cancer makes early diagnosis difficult, initial diagnosis typically occurs when the tumor has reached an advanced stage [4]. A better understanding of pancreatic carcinogenesis is urgently needed to facilitate early detection. Pancreatic intraepithelial neoplasia (PanIN) and intraductal papillary-mucinous neoplasm (IPMN) were reported to be precursor lesions of pancreatic cancer [5-8]. Development of invasive ductal adenocarcinoma has been proposed to occur via two pathways [9-11], the PanIN-Invasive ductal carcinoma (IDC) progression pathway and the IPM adenoma (IPMA)-invasive IPM carcinoma (IPMC) pathway, although some specific subtypes of IPMN, such as intestinal-type IPMN, may not progress to invasive carcinoma through the same genetic pathway as PanIN. Longnecker *et al *[12] reported that PanIN-1 and IPMA showed mild dysplasia (grade 1), PanIN-2 and IPM borderline (IPMB) lesions showed moderate dysplasia (grade 2), and PanIN-3 and IPMC (carcinoma *in situ *[CIS]) showed severe dysplasia (grade 3).

LIM-only 4 (LMO4) is one of the four members (LMOs 1, 2, 3 and 4) of the LIM-only subfamily of LIM domain proteins. LIM domains are an approximately 55-amino acid, cysteine-rich, zinc-binding motif that mediate protein-protein interactions present in a variety of proteins including LIM homeobox proteins [13]. The nuclear LIM-only proteins (LMOs 1–4) lack a DNA-binding domain but still function as transcriptional regulators by recruiting other protein partners including transcription factors [14,15]. Kenny *et al *[13] reported the isolation and characterization of *LMO4*, a novel LIM-only gene that is highly expressed in the T-lymphocyte lineage, cranial neural crest cells, somites, dorsal limb bud mesenchyme, motor neurons and Schwann cell progenitors. As well as its role in development, there are several lines of evidence suggesting that LMO4 may have roles in oncogenesis [16]. LMO4, initially described as a human breast tumor autoantigen [17], was reported to have a role in maintaining proliferation of mammary epithelium and suggested that deregulation of this gene may contribute to breast tumorigenesis [18]. Additionally, Sum *et al *[19] found that LMO4 interacts with the cofactor CtIP and the tumor suppressor breast cancer 1 (BRCA1), and inhibits the transcriptional activity of BRCA1 in both yeast and mammalian cells by functional assays. They concluded that deregulation of LMO4 in breast epithelium directly contributes to breast neoplasia by altering the rate of cellular proliferation and promoting cell invasion. In 2005, Sum and colleagues reported that *LMO4 *mRNA was overexpressed in 5 of 10 human breast cancer cell lines; *in situ *hybridization analysis of 177 primary invasive breast carcinomas revealed overexpression of *LMO4 *in 56% of the specimens [20]. It has also been reported that expression of *LMO4 *is up-regulated at the invasive front of oral cancer, suggesting a role in cancer cell invasion [21]. It was recently reported that the bone morphogenic protein (*BMP7*) gene, which controls cell proliferation and apoptosis of mammary epithelial cells, is a direct target of *LMO4 *[22]. Both pancreatic cancer and breast cancer are known to have an epithelial origin while pancreatic cancer also reveals papillo-tubular structures that are similar to the histological characteristics of the initial breast cancer [23]. As well, known genetic changes in pancreatic cancer often involve the same genes as those found in breast cancer [24]. Taken together, these findings suggest that LMO4 has critical functions in pancreatic carcinogenesis as well as in normal development. Thus clarification of the role of LMO4 may be useful for diagnosis and/or treatment of pancreatic cancer. However, little is known about the role of *LMO4 *in pancreatic cancer and carcinogenesis.

To determine whether LMO4 is correlated with pancreatic cancer and carcinogenesis, we quantified *LMO4 *mRNA levels in cultured pancreatic cell lines, bulk tissues and microdissection-based target cells (including normal pancreatic ductal, PanIN-1B and PanIN-2, IDC, IPMA, IPMB and IPMC cells), by quantitative real-time reverse transcription-polymerase chain reaction (qRT-PCR). Our goal was to characterize *LMO4 *expression in the early and late stages of pancreatic carcinogenesis to clarify both if and when overexpression of *LMO4 *occurs.

## Materials and methods

### Cultured cells

Fourteen pancreatic cancer cell lines, AsPC-1, KP-1N, KP-2, KP-3, PANC-1, SUIT-2 (provided by Dr. H. Iguchi, National Shikoku Cancer Center, Matsuyama, Japan), MIA PaCa-2 (Japanese Cancer Resource Bank, Tokyo, Japan), NOR-P1 (established in our laboratory), CAPAN-1, CAPAN-2, CFPAC-1, H48N, HS766T and SW1990 (American Type Culture Collection, Manassas, VA, USA), the HPDE cell line and six primary cultures of fibroblasts derived from pancreatic tumors were studied. Cells were maintained as described previously [25].

### Pancreatic tissues

**Figure 1:**
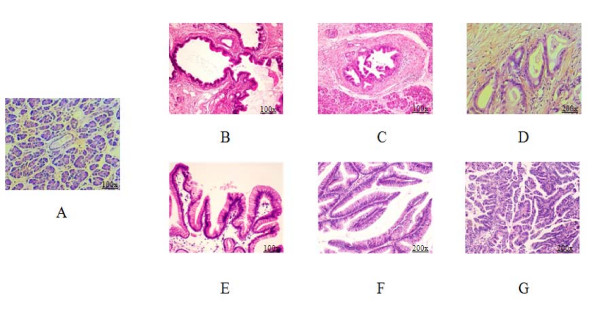
**Histologic examination of formalin-fixed, paraffin-embedded (FFPE) samples with hematoxylin and eosin (H&E) staining**. The stages of the PanIN-IDC pathway are shown as normal pancreatic ductal epithelia (A), pancreatic intraepithelial neoplasm-1B (PanIN-1B) (B), PanIN-2 (C) and invasive ductal carcinoma (IDC) (D). The stages of the intraductal papillary mucinous adenoma (IPMA)-intraductal papillary mucinous carcinoma (IPMC) pathway are shown as IPM adenoma (E), IPM borderline (F) and IPM carcinoma (G). (Magnifications of A to G are 100×, 100×, 100×, 200×, 100×, 200×, 200×, respectively).

Tissue samples were obtained during surgery at Kyushu University Hospital (Fukuoka, Japan) as described previously [26]. In brief, tissue samples were removed and divided into at least three bulk tissue specimens. The first sample was immediately but temporarily preserved in cold PBS and then embedded in OCT compound (Sakura Findek, Tokyo, Japan), snap-frozen for microdissection within 1 hour after resection, and stored at -80°C until use. The second sample was fixed in formalin, embedded in paraffin and cut into 4-μm-thick sections for hematoxylin and eosin (H&E) staining. The third sample was immediately snap-frozen for bulk tissue analysis and stored at -80°C until use. Tissues adjacent to the specimens were examined histologically and the diagnosis confirmed by two pathologists, Dr Hiroshi Yamaguchi and Dr Kohei Nakata (Figure 1). Written informed consent was obtained from all patients, and the study was approved by our institution's surveillance committee and conducted according to the Helsinki Declaration.

### RNA isolation

Total RNA was extracted from cultured cells with a High Pure RNA Isolation Kit (Roche, Mannheim, Germany). Total RNA was extracted from bulk tissues with an RNeasy Mini Kit (Qiagen, Tokyo, Japan) following the manufacturer's protocol. Total RNA was extracted from cells isolated by microdissection with the standard acid guanidinium thiocyanate-phenol-chloroform protocol [27] with or without glycogen (Funakoshi, Tokyo, Japan) [26].

### Quantitative assessment of *LMO4 *level by real-time RT-PCR

Quantitative real-time RT-PCR was performed with a QuantiTect SYBR Green RT-PCR Kit (Qiagen, Tokyo, Japan) with a Chromo4™ System (Bio-Rad, Hercules, CA, USA). In brief, the reaction mixture was first incubated at 50°C for 30 min to allow for reverse transcription. PCR was initiated with one cycle of 95°C for 15 min to activate the modified Taq polymerase followed by 40 cycles of 94°C for 15 sec, 55°C for 20 sec, and 72°C for 10 sec, and one cycle of 95°C for 0 sec, 65°C for 15 sec and +0.1°C/sec to 95°C for melting analysis. Each sample was run in triplicate. The level of *LMO4 *mRNA expression was calculated from a standard curve constructed with total RNA from the SUIT-2 pancreatic cancer cell line. The range of threshold cycles was from 20–35 cycles for *LMO4 *primers (forward, 5'-GGA CCG CTT TCT GCT CTA TG-3'; reverse, 5'-AAG GAT CAT GCC ACT TTT GG-3'), and from 7–35 cycles for *18S *rRNA primers (forward 5'-GAT ATG CTC ATG TGG TGT TG-3'; reverse, 5'-AAT CTT CTT CAG TCG CTC CA-3'). Expressions of *LMO4 *mRNA were normalized to that of *18S *rRNA.

### Microdissection-based quantitative analysis of *LMO4 *mRNA

**Figure 2:**
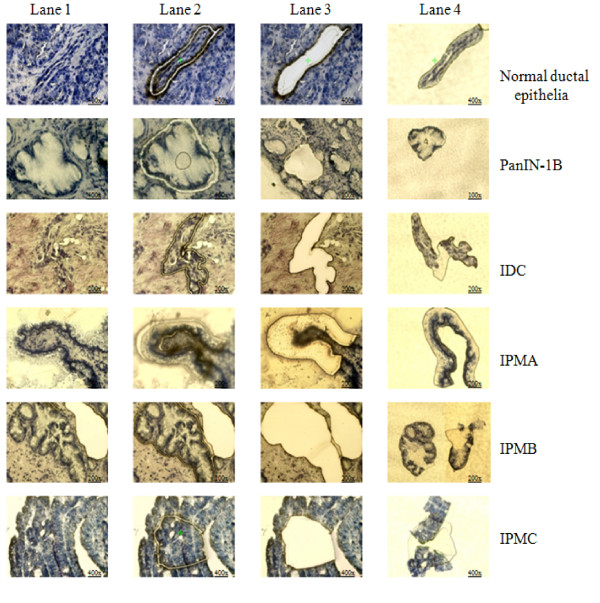
**Pictures of pancreatic normal ductal epithelia, PanIN-1B, IDC, IPMA, IPMB and IPMC lesions stained with 1% toluidine blue**. Lanes 1 to 4 show the process of microdissecting the target cells. Before cutting (lane 1), after cutting (lane 2), rest of region (lane 3) and target cells in capture (lane 4). (The magnifications are shown on the pictures).

Frozen tissues were cut into 8-μm-thick sections. IDC cells from 8 lesions, PanIN-2 cells from 3 lesions, PanIN-1B cells from 14 lesions, normal ductal epithelial cells from 13 ducts, IPMA cells from 13 lesions, IPMB cells from 15 lesions, and IPMC cells from 8 lesions, including 3 non-invasive IPMC lesions (CIS) and 5 invasive IPMC lesions, were selectively isolated with a laser microdissection and pressure catapulting system (P.A.L.M. Microlaser Technologies, Bernried, Germany) in accordance with the manufacturer's protocols (Figure 2) [28]. After microdissection, total RNA was extracted from the selected cells and subjected to qRT-PCR for quantitative measurement of *LMO4 *as described previously [26].

### Statistical analysis

Data were analyzed with multiple comparison in ANOVA (analysis of variance) and Bivariate Correlations with Statistics Package for Social Science (SPSS) software (SPSS Inc. Chicago, IL, USA) after Kolmogorov-Smirnov test to assure that each data set is showed a normal distribution. For multiple comparisons by ANOVA, we used the least significant difference (LSD) test and set the statistical significance at *p *< 0.05. We used Spearman test for bivariate correlations.

## Results

### Quantitative analysis of *LMO4 *expression in 14 pancreatic cancer cell lines, a non-neoplastic ductal epithelial cell line and six primary cultures of pancreatic fibroblasts

**Figure 3:**
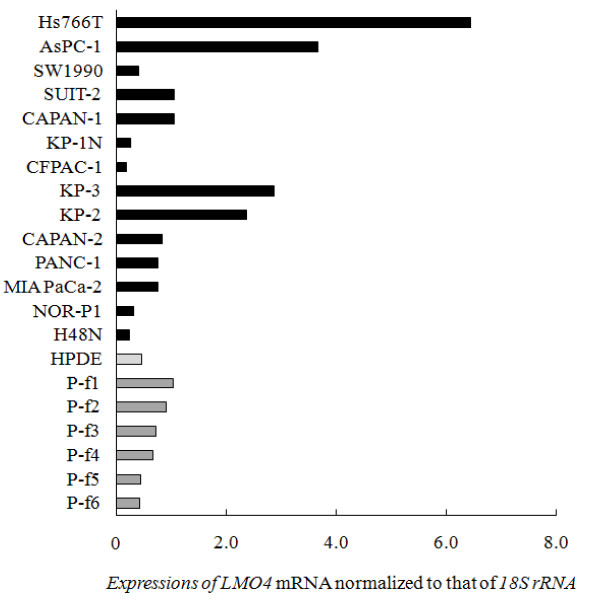
***LMO4 *expressions in pancreatic cancer cell lines, a human pancreatic ductal epithelial (HPDE) cell line and primary fibroblasts derived from pancreatic tumors**. Hs766T, AsPC-1, SUIT-2, CAPAN-1, KP-3, KP-2, CAPAN-2, PANC-1 and MIA PaCa-2 cells expressed significantly higher levels of *LMO4 *(median, 1.07) than did the HPDE cells (median, 0.46). All 6 cultures of primary fibroblasts express moderate levels of *LMO4 *(median, 0.70). The difference in *LMO4 *expression between the metastasis group (Hs766T, AsPC-1, SW1990, NOR-P1, SUIT-2 and CAPAN-1; median = 1.06) and the non-metastasis group (KP-2, KP-3, CAPAN-2, PANC-1, MIA PaCa-2 and H48N; median = 0.76) is not statistically significant (*p *= 0.75). Expression of *LMO4 *mRNA was normalized to that of *18S *rRNA.

We investigated *LMO4 *mRNA expression in 14 pancreatic cancer cell lines, HPDE, a normal pancreatic ductal cell line immortalized by transduction with the E6/E7 genes of human papillomavirus 16 [29,30], and 6 primary cultures of pancreatic fibroblasts derived from resected pancreatic tumors. As shown in Figure 3, 9 of the 14 pancreatic cancer cell lines expressed higher levels of *LMO4 *than did HPDE. We also found that all 6 primary cultures of pancreatic fibroblasts expressed moderate levels of *LMO4*. Four pancreatic cancer cell lines, Hs766T, AsPC-1, KP-2 and KP-3, expressed higher levels of *LMO4 *than did any of the primary cultures of pancreatic fibroblasts. We next examined whether *LMO4 *expression is related to the origin of these cancer cell lines, but found no correlation between *LMO4 *expression and site of origin, such as primary or metastatic tumors (Figure 3).

### Quantitative analyses of *LMO4 *expression in bulk pancreatic tissues

**Figure 4:**
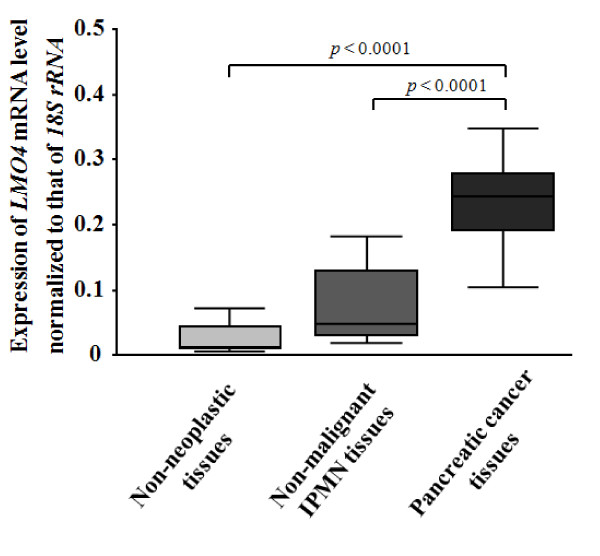
***LMO4 *expressions in bulk pancreatic tissues, including 20 non-neoplastic tissues, 11 non-malignant IPMN tissues and 11 pancreatic cancer tissues**. The mean *LMO4 *expression value for pancreatic cancer samples is 0.24 (95% CI; 0.16 – 0.32), which is higher than the *LMO4 *level of non-malignant IPMN (mean, 0.08; 95% CI, 0.04 – 0.12) or non-neoplastic tissues (mean, 0.03; 95% CI, 0.02 – 0.04). The difference in *LMO4 *expression between non-malignant IPMN and non-neoplastic tissues is not statistically significant (*p *= 0.053). The bottom and the top edges of the box mark the lower bound and upper bound of the 95% Confidence Interval for the Mean, respectively. The center horizontal line is drawn at the sample mean. The center vertical lines drawn from the boxes extend to the minimum and the maximum. Expression of *LMO4 *mRNA was normalized to that of *18S *rRNA.

In the bulk tissue analyses, we measured *LMO4 *expression in pancreatic cancer tissues (*n *= 11), and non-neoplastic tissues (*n *= 20), normal pancreatic or chronic pancreatitis-related tissues, and non-malignant IPMN tissues (*n *= 11). As shown in Figure 4, *LMO4 *expression was highest in the pancreatic cancer tissues with a mean of 0.24 (95% confidence interval [CI], 0.16 – 0.32), whereas the *LMO4 *expression levels were 0.03 (95% CI, 0.02 – 0.04) in non-neoplastic tissues and 0.08 (95% CI, 0.04 – 0.12) in non-malignant IPMN tissues. The mean *LMO4 *mRNA level in pancreatic cancer tissues was eight-fold higher than that in non-neoplastic tissues (*p *< 0.0001) and three-fold higher than that in non-malignant IPMN tissues (*p *< 0.0001). The mean *LMO4 *mRNA level in non-malignant IPMN tissues was 2.7-fold higher than that in non-neoplastic tissues, although the difference was not statistically significant (*p *= 0.053). All data from bulk tissue analyses indicated that *LMO4 *was overexpressed in pancreatic cancer.

### Microdissection-based quantitative analysis of *LMO4 *expression in IDC, PanIN-2, PanIN-1B and normal ductal cells

In general, bulk pancreatic tissues are composed of a various types of cells, including ductal epithelial, acinar, islet and mesenchymal cells, and fibroblasts. Cancer cells comprise only 30% – 70% of the cells in bulk tissue specimens of pancreatic cancer [27]. Premalignant cells, such as PanINs, and normal ductal cells comprise even smaller percentages of the cells in non-malignant tissues. The results of our present analyses of cultured cells suggested that *LMO4 *was expressed in pancreatic fibroblasts (Figure 3). Therefore, to avoid the influence of contaminating non-ductal cells, we used a laser-microdissection (LMD) method to select specific ductal cells for further analysis.

**Figure 5:**
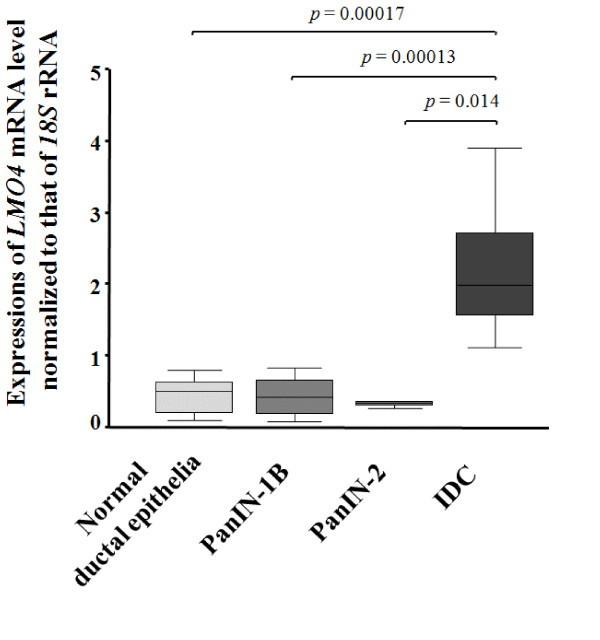
***LMO4 *expressions in the PanIN-IDC pathway, including 13 normal ductal epithelial ducts, 14 PanIN-1B lesions, 3 PanIN-2 lesions, and 8 IDC lesions**. IDC cells (mean, 2.19; 95% CI, 1.43 – 2.94) expressed significantly higher levels of *LMO4 *than did normal ductal epithelia (mean, 0.45; 95% CI, 0.30 – 0.60), PanIN-1B (mean, 0.41; 95% CI, 0.27 – 0.54), or PanIN-2 cells (mean, 0.32; 95%CI 0.18 – 0.45). The differences in *LMO4 *expression among PanIN-2, PanIN-1B, and normal ductal epithelial cells are not statistically significant (*p *= 0.54 and *p *= 0.56). The bottom and the top edges of the box mark the lower bound and upper bound of the 95% Confidence Interval for the Mean, respectively. The center horizontal line is drawn at the sample mean. The center vertical lines drawn from the boxes extend to the minimum and the maximum. Expression of *LMO4 *mRNA was normalized to that of *18S *rRNA.

To investigate the involvement of *LMO4 *in the PanIN-IDC pathway, we isolated IDC cells from 8 lesions, PanIN-2 cells from 3 lesions, PanIN-1B cells from 14 lesions and normal ductal epithelial cells from 13 ducts by LMD (Figure 2) for quantitative analysis of *LMO4 *by RT-PCR. As shown in Figure 5, IDC cells expressed significantly higher levels of *LMO4 *than did normal ductal epithelial or PanIN-1B cells (*p *< 0.0001 for both) or PanIN-2 cells (*p *= 0.014). The mean *LMO4 *expression level was 2.19 (95% CI, 1.43 – 2.94) in IDC cells, 0.32 (95% CI, 0.18 – 0.45) in PanIN-2 cells, 0.41 (95% CI, 0.27 – 0.54) in PanIN-1B cells and 0.45 (95% CI, 0.30 – 0.60) in normal ductal epithelial cells. The difference in *LMO4 *expression between PanIN-2 and normal ductal cells or between PanIN-1B and normal ductal cells was not significant (*p *= 0.54 and *p *= 0.56, respectively). These data suggested that *LMO4 *is overexpressed in pancreatic cancer, especially in the invasive step of cancer, but not in the early stage of pancreatic carcinogenesis.

### Quantitative analyses of *LMO4 *expressions in IPMC, IPMB, IPMA and normal ductal cells

**Figure 6:**
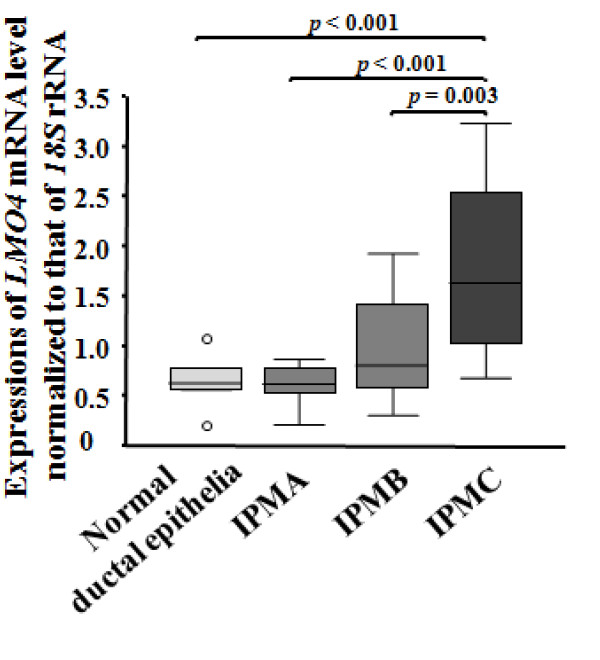
***LMO4 *expressions in the IPMA-IPMC pathway, including 6 normal ductal epithelial ducts, 13 IPMA lesions, 15 IPMB lesions and 8 IPMC lesions (5 for invasive IPMC and 5 for non-invasive IPMCs)**. IPMC cells expressed significantly higher levels of *LMO4 *(mean, 1.79; 95% CI, 0.99 – 2.58) than did IPMB (mean, 0.98; 95% CI, 0.69 – 1.27) (*p *= 0.003), IPMA (mean, 0.60; 95% CI, 0.47 – 0.73) (*p *< 0.001), or normal ductal epithelial cells (mean, 0.65; 95% CI, 0.34 – 0.95) (*p *< 0.001). The differences in *LMO4 *expression between IPMB and IPMA cells (*p *= 0.09), IPMB and normal ductal epithelial cells (*p *= 0.2) and IPMA and normal ductal epithelial cells (*p *= 0.9) are not significant. The bottom and the top edges of the box mark the lower bound and upper bound of the 95% Confidence Interval for the Mean, respectively. The center horizontal line is drawn at the sample mean. The center vertical lines drawn from the boxes extend to the minimum and the maximum. Expression of *LMO4 *mRNA was normalized to that of *18S *rRNA.

To investigate the correlation of *LMO4 *expression with the IPMA-IPMC pathway, we microdissected IPMC cells from 5 invasive lesions and 3 non-invasive lesions (CIS), IPMB cells from 15 lesions, IPMA cells from 13 lesions and normal ductal epithelial cells from 6 ducts. We then measured *LMO4 *expression in these cells by qRT-PCR (Figure 2). As shown in Figure 6, IPMC cells expressed higher levels of *LMO4 *(mean, 1.79; 95% CI, 0.99 – 2.58), than did normal ductal cells (*p *< 0.001), IPMA cells (*p *< 0.001) and IPMB cells (*p *= 0.003). There was no significant difference in *LMO4 *expression between invasive IPMC and non-invasive IPMC (CIS). Although the mean *LMO4 *expression in IPMB cells (0.98; 95% CI, 0.69 – 1.27) was higher than that in IPMA (0.60; 95% CI, 0.47 – 0.73) or normal ductal cells (0.65; 95% CI, 0.34 – 0.95), the differences in *LMO4 *expression were not significant. Taken together, these data suggested that *LMO4 *expression may be up-regulated during the late stages of carcinogenesis of IPMN.

### Quantitative analyses of *LKB1 *expressions in cultured and microdissected cells, and correlation analyses between *LKB1 *and *LMO4 *in pancreatic carcinogenesis

**Figure 7:**
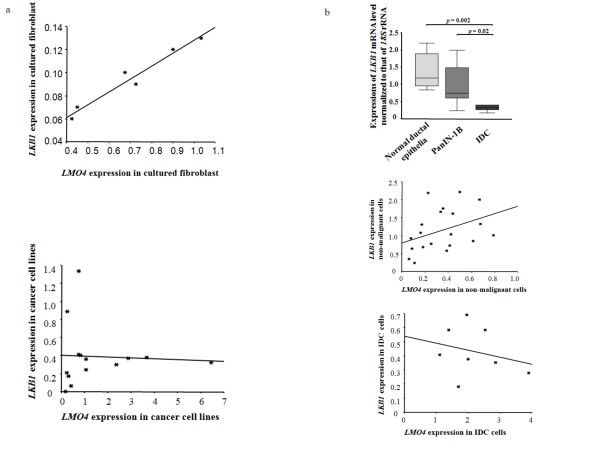
**Correlation analyses between *LKB1 *and *LMO4 *in pancreatic carcinogenesis**. a, Correlation between *LKB1 *and *LMO4 *in primary cultured fibroblasts (spearman test, *p *= 0.037, top) and correlation between *LKB1 *and *LMO4 *in cultured cancer cell lines (spearman test, *p *= 0.39, bottom). b, Expression of *LKB1 *mRNA levels in microdissected normal ducts, PanIN-1B cells, and IDC cells (top). The bottom and the top edges of the box mark the lower bound and upper bound of the 95% Confidence Interval for the Mean, respectively. The center horizontal line is drawn at the sample mean. The center vertical lines drawn from the boxes extend to the minimum and the maximum. Correlation between *LKB1 *and *LMO4 *mRNA levels in non-malignant cells (including normal ducts and PanIN-1B cells, spearman test, *p *= 0.042, middle), and correlation between *LKB1 *and *LMO4 *mRNA levels in IDC cells (spearman test, *p *= 0.453, bottom). Expression of *LMO4 *mRNA was normalized to that of *18S *rRNA.

LKB1 (also called STK11) is a tumor suppressor gene in Peutz-Jeghers syndrome [31]. Loss of this gene is found in pancreatic cancer [32,33]. Recently, LKB1 was reported to induce p21 expression in collaboration with LMO4 [34]. To investigate the potential role of *LMO4 *in pancreatic carcinogenesis, we measured *LKB1 *mRNA expression in cultured and microdissected cells and investigated any correlation between *LKB1 *and *LMO4 *expression. In the analysis of cultured cells, we found significant correlation between *LKB1 *and *LMO4 *mRNA levels in primary cultured fibroblasts (n = 6, spearman test, *p *= 0.024; Figure 7a, top). By contrast, there was no significant correlation between *LKB1 *and *LMO4 *mRNA levels in cultured cancer cell lines (n = 14, spearman test, *p *= 0.33; Figure 7a bottom). In the analysis of microdissected cells, IDC cells expressed significantly lower levels of *LKB1 *mRNA than did PanIN-1B or normal ductal cells (n = 12, *p *= 0.02; n = 8; *p *= 0.002, respectively; Figure 7b top). There was a significant correlation between *LKB1 *and *LMO4 *mRNA levels in non-malignant cells, including normal pancreatic ductal epithelia and PanIN-1B cells (n = 20, *p *= 0.042, Figure 7b middle), but no significant correlation in IDC cells (n = 8, *p *= 0.45, Figure 7b bottom).

## Discussion

In the present study, we performed quantitative real-time RT-PCR to measure *LMO4 *expression during pancreatic carcinogenesis through the PanIN-IDC and IPMA-IPMC pathways. Analyses of cultured cells revealed that 9 of 14 pancreatic cancer cell lines and all primary cultures of pancreatic fibroblasts expressed higher levels of *LMO4 *than did a non-neoplastic pancreatic ductal cell line. Bulk tissue analysis showed that pancreatic cancer tissues expressed higher levels of *LMO4 *than non-neoplastic and non-malignant IPMN tissues; however, the difference in *LMO4 *expression between non-neoplastic tissues and non-malignant IPMN was not significant. To avoid the influence of *LMO4*-expressing non-ductal cells contained in bulk tissues, we microdissected target cells, such as IDCs, PanINs, IPMNs and non-neoplastic ductal cells, and measured *LMO4 *expression in the microdissected cells. It is usually difficult to obtain frozen sections of intermediate or high-grade PanIN-2 or PanIN-3 (CIS) or non-invasive IPMC (CIS) lesions. In the present study, we obtained frozen sections from 3 cases of PanIN-2 lesions and 3 of non-invasive IPMC. We found that the *LMO4 *expression in IDC cells was significantly higher than those in PanIN-1B, PanIN-2, and normal ductal cells; however, the PanIN-2 sample number was small. We also found that both invasive and non-invasive IPMC cells expressed higher levels of *LMO4 *than did non-malignant IPMN or normal ductal cells. By contrast, we could not detect any differences in *LMO4 *expression among PanIN-1B, PanIN-2 and normal ductal cells, or among IPMA, IPMB, and normal ductal cells. Taken together, these data suggested that *LMO4 *expression is up-regulated in pancreatic cancer but not in low-grade intraductal precursors in both the PanIN-IDC and IPMA-IPMC pathways.

This is the first report to use qRT-PCR for analyses of *LMO4 *expression during pancreatic carcinogenesis. *LMO4 *is reported to have an oncogenic role in carcinogenesis and in carcinoma progression in breast cancer and SCC [18-21]. However, the human *LMO4 *gene is located on chromosome 1p22.3 [35], which is a region deleted in several human cancers, such as those of liver, skin, and lung [36,37], and Setogawa *et al *reported that the tumor suppressor LKB1 induces p21 expression in collaboration with LMO4, suggesting that LMO4 may have a tumor suppressor function [34]. In the present study, we found that there was significant correlation between *LMO4 *and *LKB1 *in both primary cultured fibroblasts and microdissected non-malignant cells, but there was not a significant correlation between *LMO4 *and *LKB1 *in cancer cells. We also found the downregulation of *LKB1 *mRNA in IDC cells, consistent with LKB1's tumor suppressive function. Our data suggest that alteration of the LKB1-LMO4 balance is involved in pancreatic carcinogenesis, although the exact function of LMO4 in pancreatic carcinogenesis remains unknown. Taken together, there appears to be a conflicting function of LMO4 in carcinogenesis as a tumor suppressor or as an oncogene; it is reasonable that LMO4, a transcription regulator, may have multiple functions in individual cancers, like E2F1, an another transcription factor, that was reported both as an oncogene by stimulating cell proliferation [38] and as a tumor suppressor by signaling p53-dependent apoptosis [39].

Recently, Murphy *et al *[40] used immunohistochemical staining and reported that a subset of patients with low LMO4 expression-pancreatic cancers had poor outcomes. In the present study, *LMO4 *mRNA was not overexpressed in any of the 14 pancreatic cancer cell lines. However, all IDC cells microdissected from cancer tissues showed relatively high expression of *LMO4 *mRNA although the sample number was small. This might have been the result of using of frozen sections with a histological diagnosis of moderately or well-differentiated adenocarcinoma, which can be microdissected easily. Patients with well-differentiated adenocarcinoma usually have better prognosis [41]; thus the present data may be partially consistent with Murphy's result demonstrating that high LMO4-pancreatic cancers are associated with a significant survival advantage for patients with surgical resection.

Taken together, it remains unclear if LMO4 has an oncogenic function or a tumor suppressive function in pancreatic carcinogenesis. To better clarify the functional roles of LMO4 in pancreatic carcinogenesis, further examinations such as inhibition experiments using RNAi technology are needed.

In conclusion, the present results showed that *LMO4 *is overexpressed in pancreatic cancer related to both the PanIN-conventional IDC pathway and the IPMA-IPMC pathway, but not at the early stages of pancreatic carcinogenesis.

## Competing interests

The authors declare that they have no competing interests.

## Authors' contributions

JY participated in the design of the study, carried out the RNA expression analysis, Laser Capture Microdissection studies, statistical analysis, and drafted the manuscript. KO and KM participated in the design and coordination of the study, performed the statistical analysis, and critically revised the manuscript. LC performed realtime expression analysis and critically revised the manuscript. KN and HY participated in histologic examination and critically revise the manuscript. HF, TE and HK performed the statistical analysis and critically revised the manuscript. MT planned and coordinated the study, and critically revised the manuscript. All authors have read and approved the final version of the manuscript.
